# A Comparative Study for the Prediction of the Compressive Strength of Self-Compacting Concrete Modified with Fly Ash

**DOI:** 10.3390/ma14174934

**Published:** 2021-08-30

**Authors:** Furqan Farooq, Slawomir Czarnecki, Pawel Niewiadomski, Fahid Aslam, Hisham Alabduljabbar, Krzysztof Adam Ostrowski, Klaudia Śliwa-Wieczorek, Tomasz Nowobilski, Seweryn Malazdrewicz

**Affiliations:** 1 Department of Civil Engineering, Abbottabad Campus, COMSATS University Islamabad, Abbottabad 22060, Pakistan; furqan@cuiatd.edu.pk; 2 Faculty of Civil Engineering, Cracow University of Technology, 24 Warszawska Str., 31-155 Cracow, Poland; krzysztof.ostrowski.1@pk.edu.pl (K.A.O.); klaudia.sliwa-wieczorek@pk.edu.pl (K.Ś.-W.); 3 Department of Materials Engineering and Construction Processes, Wroclaw University of Science and Technology, Wybrzeże Wyspiańskiego 27, 50-370 Wroclaw, Poland; pawel.niewiadomski@pwr.edu.pl (P.N.); tomasz.nowobilski@pwr.edu.pl (T.N.); seweryn.malazdrewicz@pwr.edu.pl (S.M.); 4 Department of Civil Engineering, College of Engineering in Al-Kharj, Prince Sattam Bin Abdulaziz University, Al-Kharj 11942, Saudi Arabia; f.aslam@psau.edu.sa (F.A.); h.alabduljabbar@psau.edu.sa (H.A.)

**Keywords:** self-compacting concrete, fly ash, machine learning, artificial neural network, gene engineering programming

## Abstract

Artificial intelligence and machine learning are employed in creating functions for the prediction of self-compacting concrete (SCC) strength based on input variables proportion as cement replacement. SCC incorporating waste material has been used in learning approaches. Artificial neural network (ANN) support vector machine (SVM) and gene expression programming (GEP) consisting of 300 datasets have been utilized in the model to foresee the mechanical property of SCC. Data used in modeling consist of several input parameters such as cement, water–binder ratio, coarse aggregate, fine aggregate, and fly ash (FA) in combination with the superplasticizer. The best predictive models were selected based on the coefficient of determination (*R*^2^) results and model validation. Empirical relation with mathematical expression has been proposed using ANN, SVM, and GEP. The efficiency of the models is assessed by permutation features importance, statistical analysis, and comparison between regression models. The results reveal that the proposed machine learning models achieved adamant accuracy and has elucidated performance in the prediction aspect.

## 1. Introduction

In recent years, concrete technology has been improving due to the fact that it is the most commonly used building material in the world. The knowledge of advance techniques of designing concrete has also improved recently due to different type of concrete being designed containing different admixtures [1]. One of the results of developing concrete designing technology is self-compacting concrete (SCC) [2]. Self-compacting concrete is defined as a cementitious material that can flow under its own weight and was first developed in the late 1990s in Japan. SCC deforms efficiently and shows maximum resistance to segregation and bleeding as per American Concrete Institute committee 237 R-07 [3]. Moreover, due to its workability, SCC is more often used where there is a need of creating different shapes of the elements or there are some parts of elements hardly reachable [4].

SCC is an advanced material with similar strength and durability as compared with traditional vibrated concrete; however, very often, due to self-venting, it is characterized by better performance; thus, it is sometimes used in strengthening of reinforced concrete (RC) beams [5,6]. Even though the SCC is commonly used in construction practice, designing proper mixture of SCC is still a difficult task to solve. The main reason of this is the fact that concrete itself is a quasi-brittle material [7]; the SCC requires relevant flow, and more often, industrial wastes are added as by-products: fly ash (FA), silica fume (SF), and ground granulated blast furnace slag (GGBFS) [8]. There are certain waste byproducts that, when included in the cementitious system as a partial replacement of cement, substantially reduce the desired energy and CO_2_ emission [9].

In light of sustainable development and waste management, investigating the influence of the addition of the aforementioned by-products on cementitious composites properties should not be neglected. Fly ash is a fine-grained dust, consisting mainly of spherical, vitrified grains obtained by burning pulverized coal with or without co-incineration, showing pozzolanic properties and mainly containing Al_2_O_3_ and SiO_2_. Fly ash may be used in the production of concrete if it meets the requirements included in the standards [10]. The use of fly ash in concrete brings many benefits, such as: completing the particle size distribution curve, increasing the final strength of concrete as a result of the pozzolanic activity of fly ash, compacting the microstructure, and easier displacement of aggregate grains in relation to each other, which is the essence of a fresh self-compacting concrete mixture [11].

The SCC’s complex structure requires a rigorous mixed design process for achieving its required properties. The SCC mixture can differ, when analyzing the literature, due to variations in the quantity and quality of mineral admixtures, as well as design standards. What is more, the general relationship between the binder ratio to mineral admixtures, chemical admixtures, w/b ratio, and aggregate particle size seems to be ambiguous. Meanwhile, traditional methods were used by many researchers in achieving SCC properties, but the modeling aspect and optimization of mineral admixture are still missing in most aspects [12].

Computation methods as well as machine learning techniques have recently become a powerful way of modeling and estimating an extensive series of problems, particularly in modeling concrete properties [13,14,15]. Numerous studies have been conducted in the prediction of mechanical properties of self-compacting concrete and some selected of the latest, together with the name of the applied machine learning algorithm and used waste material, are listed in Table 1.

Despite the fact that researchers are implementing machine learning algorithms in concrete investigations, there is a lack of works focusing on models predicting the compressive strength of SCC modified with FA using comparative analyses containing artificial neural networks (ANN), support vector machine (SVM) and gene expression programming (GEP) combined. Taking into account the information presented in the Table 1, there is lack of comparative analysis of different machine learning algorithms used for self-compacting concrete compressive strength prediction. Thus, the aim of this study is to perform such analysis using ANN, SVM, and GEP and also to compare the obtained results with similar scientific works presented in the literature.

## 2. Research Significance

The novelty of this research is the usage of the newest machine learning algorithms in the comparative manner in order to evaluate the compressive strength of fly ash-based self-compacting concrete. For this purpose, the artificial neural network, support vector machine, and genetic expression programming were used. In particular, the novelty of this research is the usage of the genetic expression programming for this purpose. The best model among those investigated was selected after optimization. Permutation features and statistical analysis with in-depth error measures are conducted to compare the accuracy of aforementioned models and comparing them with others in the scientific field.

## 3. Prediction Methods

### 3.1. Artificial Neural Network (ANNs)

Artificial neural networks are algorithms simulating the microstructure (neurons) of a biological nervous system [27,28,29]. Their structure is similar to the biological connection between neurons in the human brain. The ANNs consist of layers: input (consist of variables used in order to forecast the investigated property), hidden (consist of nodes connected with other layers using functions and weights) and output (which is consist of predicted variables). It is possible to analyze data using ANN thanks to learning algorithms such as: quasi-Newtons, Levenberg–Marquardt’s and conjugate gradients [8]. ANNs are widely used in many applications and can therefore be a useful tool in engineering applications [30].

**Figure 1:**
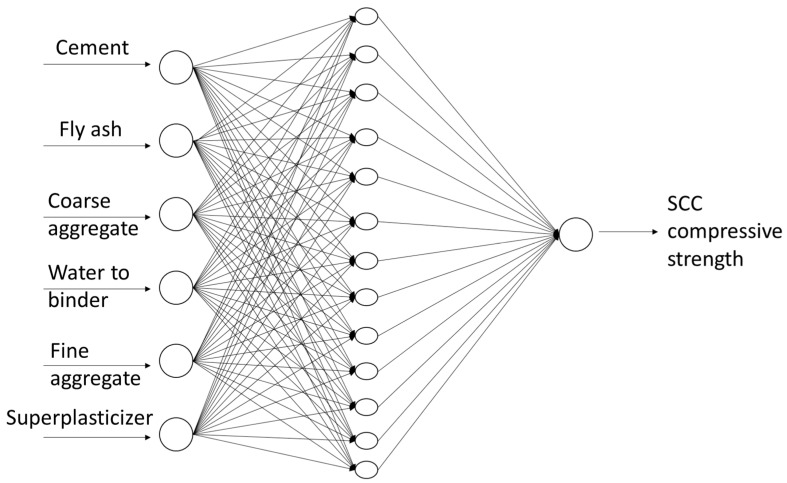
The topology of the neural network used in the study.

In this study, a multi-layer perceptron (MLP) feed-forward with backpropagation algorithm ANNs have been selected. One hidden layer and varying neuron numbers are selected to find the optimum performance of the multilayer perceptron neuron network (MLPNN) [31]. The learning algorithms used in ANNs modeling of SCC compressive strength were the Broyden–Fletcher–Goldfarb–Shano algorithm and the Levenberg–Marquardt algorithm. The data set division was fixed as: 70% of data was used in training process and 30% of data was used in processes of testing and validation [32]. Moreover, optimization of the training, validation set, and training set was obtained by changing the number of neuron layers with iteration, and vice versa. The most accurate results were obtained for the topology of six inputs, 13 hidden neurons, and one output. The topology of this network is presented in Figure 1 and described in Table 2.

### 3.2. Support Vector Machine (SVM)

**Figure 2:**
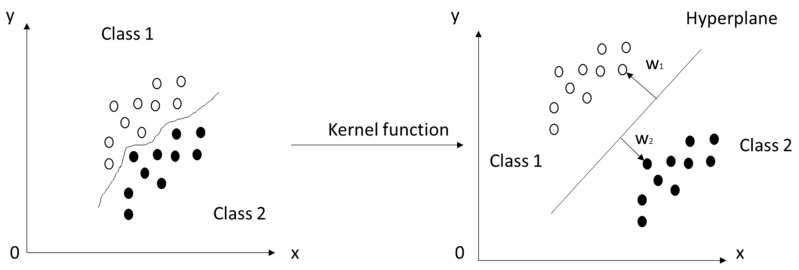
Graphical interpretation of support vector machine method.

The support vector machine is a supervised learning model used for analyzing classification and regression data, invented by Vapnik [33]. The data are represented as a map of points in space and the solution is the hyperplane (lane in 2D, plane in 3D, etc.) with the widest possible gap between two classes. Each point in this space is described with support vectors; however, there are some situations wherein the division of the data set is possible only after using kernel functions, presented in Figure 2.

The support vector machine has been successfully used in solving some engineering problems, e.g., analyzing the durability of lightweight cement composites with hydrophobic coatings modified by nanocellulose [33]. In this work, the v-SVM was used, with linear kernel function as the most accurate. The other kernel function tested: polynomial, RBF, or sigmoid were not that significantly accurate.

### 3.3. Genetic Engineering Programming (GEP)

**Figure 3:**
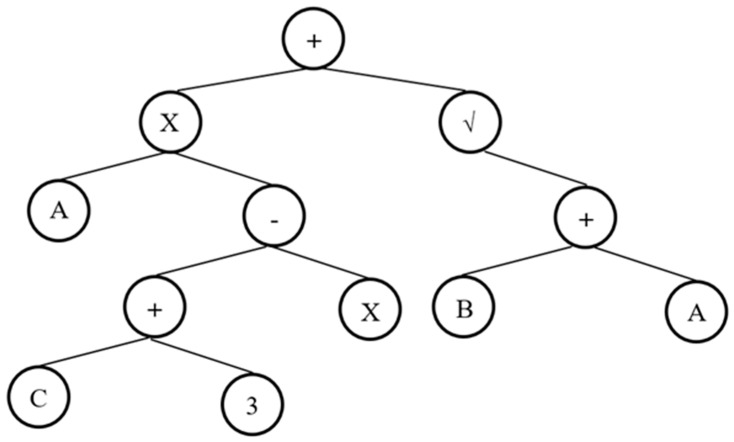
An example of tree expression (ETs).

Genetic engineering programming is a versatile approach as it incorporates both gene algorithms (GAs) and genetic programming (GP) [34]. This algorithm consisting of trees, that are called expression trees (ETs), and the benefit of this solution is the fact of adamantly simplified at the chromosome level operation of genetic work [35]. Another modification in GEP, in comparison to GAs, is that the individual chromosomes that contain numerous genes and are additionally classified into the model head and tail [36]. Each individual gene of GEP, presented as a node of the ET, stores a number of variables with constant length, function set, and terminal sets. Function set, terminal set, and variables are connected with each other via a linear genetic code. It is worth mentioning here that these sets must have closure property. A sample of the GEP gene can also be represented by an expression tree (ETs) diagram. An example of ET diagram is shown in Figure 3.

**Figure 4:**
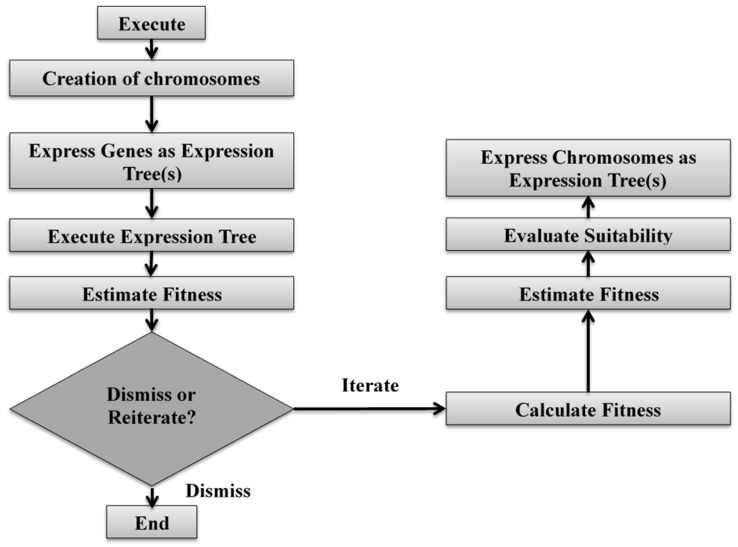
Schematic diagram of GEP algorithm.

It is expected that every gene (chromosome) contains the head, which executes the algorithm by creating chromosomes. The individuals (gene) in GEP are selected and represented as expression tree(s) with the execution of the analysis. After performing the analysis, the fitness is estimated; based on this, the decision of dismissing or reiterating is made. Dismissing the fitness finishes the algorithm, while, during reiteration, the fitness is calculated and estimated once again in order to evaluate the suitability for another expression of chromosomes as expression trees. The schematic diagram of the GEP algorithm is shown in Figure 4.

## 4. Data Presentation

### 4.1. Correlation Graph Python Programming Based

**Figure 5:**
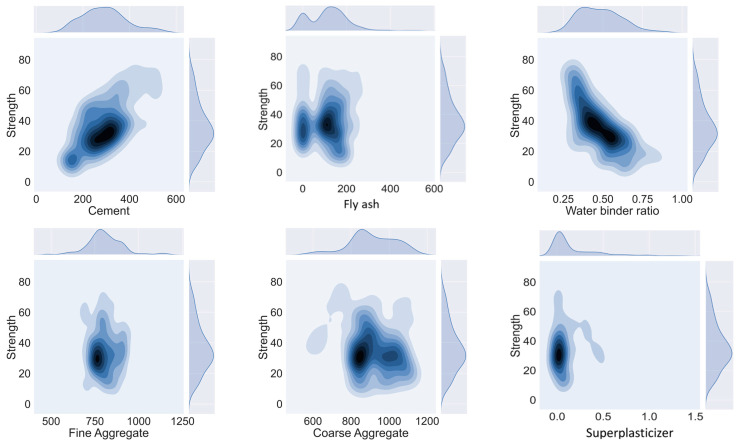
Concentration of input variables in achieving maximum compressive strength of SCC.

The collected SCC database taken from published literature [17,37,38,39,40,41,42,43,44,45,46,47,48,49,50,51,52,53,54,55,56,57,58,59,60] includes information on the water–binder ratio, fly ash, fine and coarse aggregate, superplasticizer, and cement content (see Appendix A). Each model performance is governed by the distribution of its parameters [61]. It can be seen that machine learning and artificial intelligence are hand full tools in the prediction of mechanical properties of SCCs. The distribution and relationship (optimal quantities) of input parameters to its output can be seen in contour form in Figure 5. It can be seen that with the increasing value of cement content, the compressive strength value has also increased; however, it is the opposite in the case the of water–binder ratio, wherein the increase of this ratio results in a decrease in compressive strength. Moreover, using these variable concentrations in SCCs yield maximum compressive strength output, thus eliminating its need for going in hit and trial methods to obtain the target strength. Furthermore, the range and description of data is shown in Table 3 and Table 4. It may be concluded that machine learning and deep learning approaches adamantly benefit in the prediction of the mechanical aspect of SCCs.

### 4.2. Sensitivity Analysis or Permutation Feature Importance

**Figure 6:**
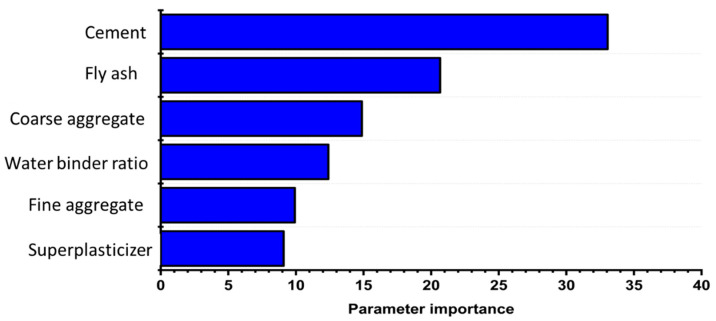
Features importance in strength prediction.

The influence of parameters on the compression strength of SCC was calculated by using machine learning (python) based program. It can be seen in Figure 6 that cement and fly ash play a vital role in SCC compressive strength prediction with 53% of their net contribution, whereas the coarse aggregate and water–binder ratio have an influence of 27.27% on the compressive strength of SCC.

Development of the SCCs model incorporating waste material is based on the selection of input parameters. These variables have an intransigent impact on SCCs mechanical properties. All parameters in the dataset were carefully studied, and only the influential parameters for a generalized relationship were selected. The compressive strength response (${f^{\prime }}_{c}$) of SCC depends upon the following factors as illustrated in Equation (1).

(1) $${f^{\prime }}_{c}=f\left(\left(\mathrm{fly}\;\mathrm{ash},\;\mathrm{SP}\right),\mathrm{Fine}\;\mathrm{aggregate},\mathrm{Coarse}\;\mathrm{aggregate},\frac{\mathrm{Water}}{\mathrm{Binder}}\right)$$

It must be noted that properly fitting parameters play an adamant part in the effectiveness and simplification of the established model. The factors for the GEP algorithm were calculated on the premise of research recommendations and numerous preliminary runs [62]. It must be kept in mind that gene chromosomes (population size) and head sizes are the key aspects in controlling program run time. Larger chromosome population and head size result in a longer time of test. Due to the number of possible results and the difficulty of the assessment model estimation, three best populations, i.e., 50, 100, or 150, and one head size were taken into consideration. The parameters for the model used in the GEP algorithm are listed in Table 5.

The correlation coefficient (*R*^2^) is a common mean degree of performance of any machine learning model. Nevertheless, the inconsiderateness of *R* to divide and multiply the productivity values into a constant implies that *R* (coefficient of relation) cannot be used exclusively as the predictive precision of any model. Therefore, errors such as the relative root mean square error (*RMSE*), mean absolute error (*MAE*), and relative mean square error (*RSE*) were also calculated. An output index or performance index (ρ) is proposed to measure model efficiency as a result of both *R* and *RRMSE* [63]. The calculated expressions are given as equations for these error functions, which are listed below:

(2) $$RMSE=\sqrt{\frac{\sum_{i=1}^{n}\;{\left(ex_{i}-mo_{i}\right)}^{2}}{n}}$$

(3) $$MAE=\frac{\sum_{i=1}^{n}|ex_{i}-mo_{i}|}{n}$$

(4) $$R=\frac{\sum_{i=1}^{n}\left(ex_{i}-{\bar{ex}}_{i}\right)\left(mo_{i}-{\bar{mo}}_{i}\right)}{\sqrt{\sum_{i=1}^{n}{\left(ex_{i}-{\bar{ex}}_{i}\right)}^{2}\sum_{i=1}^{n}{\left(mo_{i}-{\bar{mo}}_{i}\right)}^{2}}}$$

where $ex_{i}$, $mo_{i}$, ${\bar{ex}}_{i}$, and ${\bar{mo}}_{i}$ are experimental values setup and model domain.

## 5. Results and Discussion

### 5.1. Artificial Neural Network

**Figure 7:**
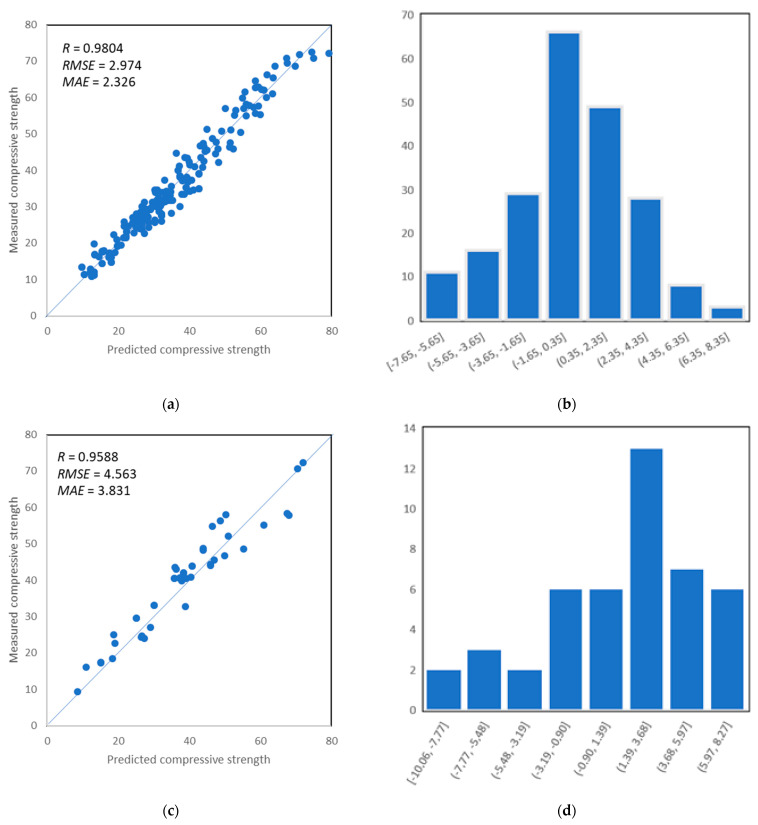

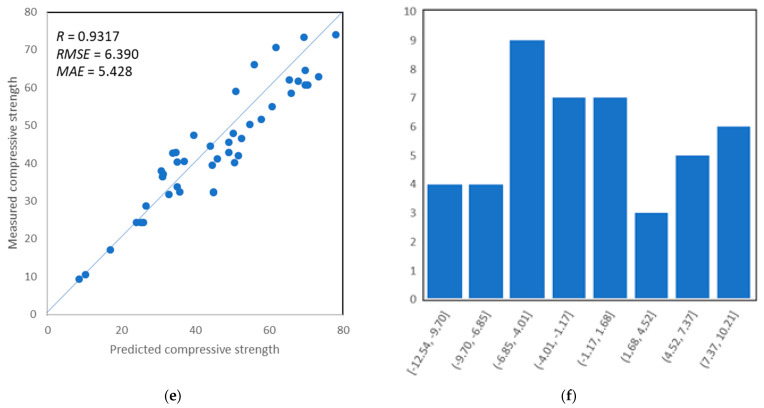
(**a**) Predictions vs. target data of training set; (**b**) error distribution of ANN model with prediction versus training set; (**c**) predictions vs. target data of validation set; (**d**) error distribution of ANN model with prediction versus validation set; (**e**) predictions vs. target data of testing set; (**f**) error distribution of ANN model with prediction versus testing set.

The influence of variables including regression coefficient *R*^2^ as well as statistical characteristics of errors between actual targets and modeled outputs are measured for the performance evaluation of the MLP-ANN model. The network output is assessed independently for training, validation, and testing set. The correlation between experimental values and prediction sets for training, validation, and testing set, respectively, are shown in Figure 7. It shows that the obstinate relation between an experimental set with modeled output for data exists. It can be seen that the training set, validation set, and test set give a coefficient of correlation close to 1, as illustrated in Figure 7a,c,e. Moreover, the prediction accuracy by ANN can also be evaluated by its error distribution. Figure 7b,d,f, present the error distribution of the training set, validation set, and testing set with prediction to output variables, showing satisfactory performance of the model. It can be seen that the error distribution of training set data between the experimentally measured compressive strength and predicted lies mostly below 10 MPa, showing that 93% of errors between the measured values and the predicted values lie in the range of 0 MPa to 10 MPa with error values between −7.65 MPa and 8.35 MPa, respectively, for training, as depicted in Figure 7b. Similarly, validation demonstrates the same trend by showing lesser error distribution in the same range of error values between −10.06 MPa and 8.27 MPa, as illustrated in Figure 7d, and for the testing set, the range of error values was a little bit higher and ranged between −12.54 MPa and 10.21 MPa, as depicted in Figure 7f. Thus, the prediction model shows obstinate and adamant modeling in relation to prediction and experimental results.

### 5.2. Support Vector Machine

**Figure 8:**
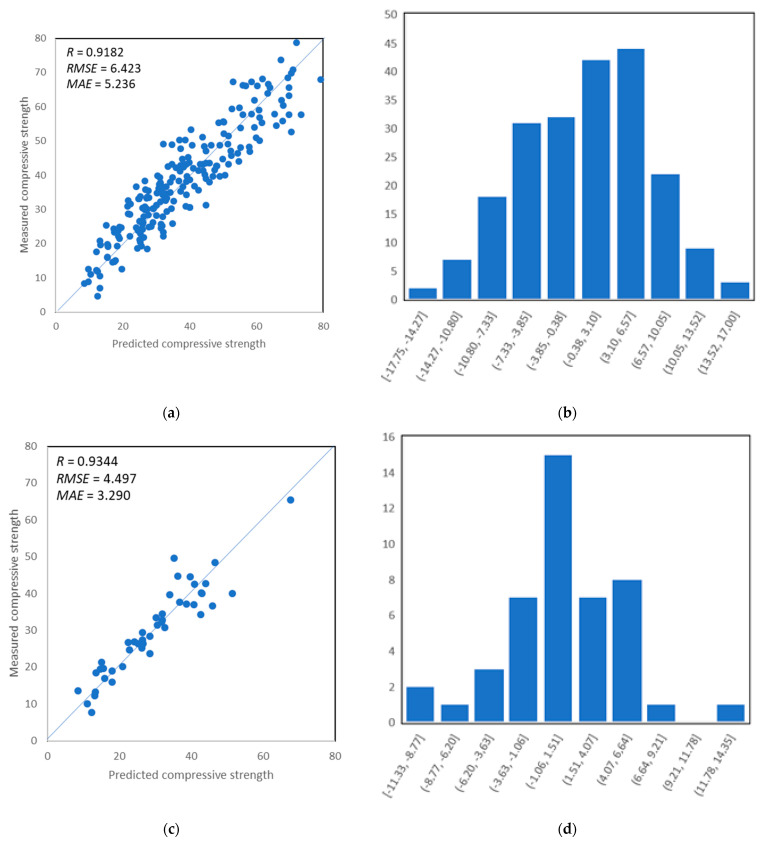

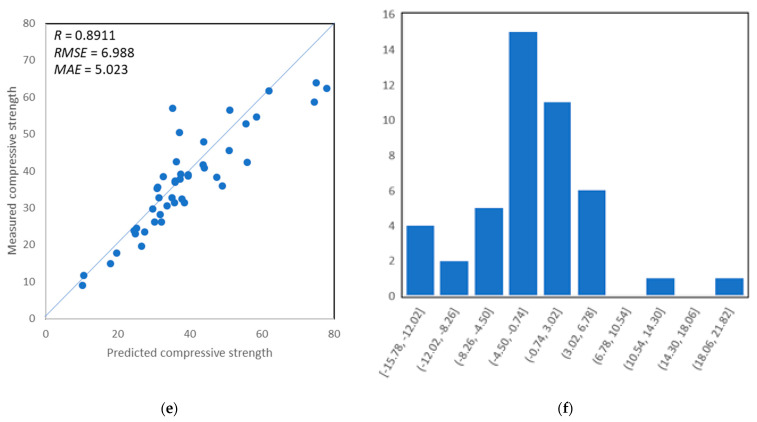
(**a**) Predictions vs. target data of training set; (**b**) error distribution of SVM model with prediction versus training set; (**c**) predictions vs. target data of validation set; (**d**) error distribution of SVM model with prediction versus validation set; (**e**) predictions vs. target data of testing set; (**f**) error distribution of SVM model with prediction versus testing set.

The influence of variables including regression coefficient *R*^2^ as well as statistical characteristics of errors between actual targets and modeled outputs are measured for the performance evaluation of the SVM model. The correlation between experimental values and prediction sets for training, validation, and testing set, respectively, are shown in Figure 8. It shows that the relation between an experimental set with modeled output for data exists, but it is not as sufficient as in comparison to ANN. It can be seen that training set, validation set, and test set give the coefficient of correlation are lower than for ANN but are still very high, as illustrated in Figure 8a,c,e. Moreover, the prediction accuracy by SVM is also illustrated by its error distribution, presented in Figure 8b,d,f). It can be seen that error values ranges between −17.75 MPa and 17.00 MPa, respectively, for training, as depicted in Figure 8b. Similarly, validation demonstrates the same trend by showing lesser error distribution in the same range of error values between −11.33 MPa and 14.35 MPa, as illustrated in Figure 8d, and for the testing set, the range of error values was a little bit higher and ranges between −15.78 MPa and 21.82 MPa, as depicted in Figure 8f. Thus, the prediction model shows less accuracy in comparison to ANN.

### 5.3. Gene Expression Programming

**Figure 9:**
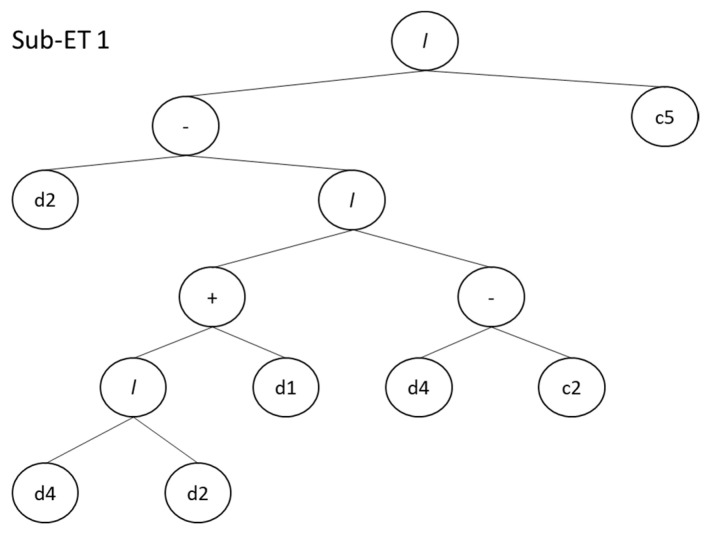

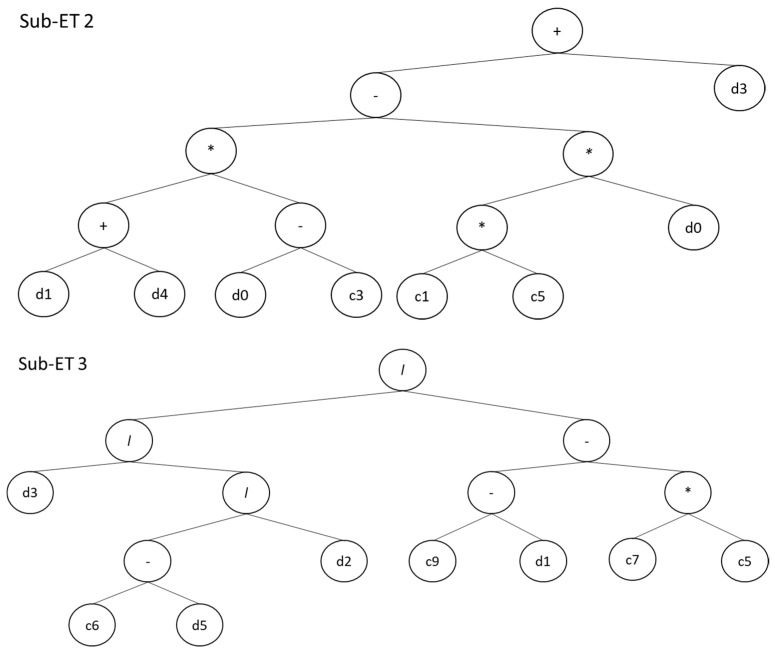
GEP model expression trees of SCC.

The output of the GEP algorithm for the SCC model is denoted as an expression tree(s), as illustrated in Figure 9. The GEP algorithm solves nonlinear expressions as well as linear ones by forming a tree-like structure, which can then be used to form an equation used to predict the model outcome. These ETs were then decoded to give empirical relationships. The ETs for compressive strength of SCC contains four basic mathematical functions containing addition, multiplication, subtraction, and division. Moreover, it can be seen that these expression trees contain parameters and constants to prepare empirical equations, as shown in Table 6.

Defined relationships between ETs and genes help in predicting the compressive properties of self-compacting concrete ($f_{c}^{\prime }$). The response to predict the compressive strength is then proposed with expression trees by using Equation (5).

(5) $$f_{c}^{\prime }=A\times B\times C$$

where:

(6) $$A=\left(d\left(2\right)-\left(\frac{\frac{d\left(4\right)}{d\left(2\right)}+d\left(1\right)}{d\left(4\right)-G1C2}\right)\right)/G1C5$$

(7) B=((((d(1)+d(4)×(d(0)−G2C3))−((G2C1×G2C5)×d(0)))+d[3])

(8) $$C=\left(\frac{\frac{d\left(3\right)}{\frac{G3C6-d\left(5\right)}{d\left(2\right)}}}{\left(G3C9-d\left(1\right)\right)-\left(G3C7\times G3C5\right)}\right)$$

**Figure 10:**
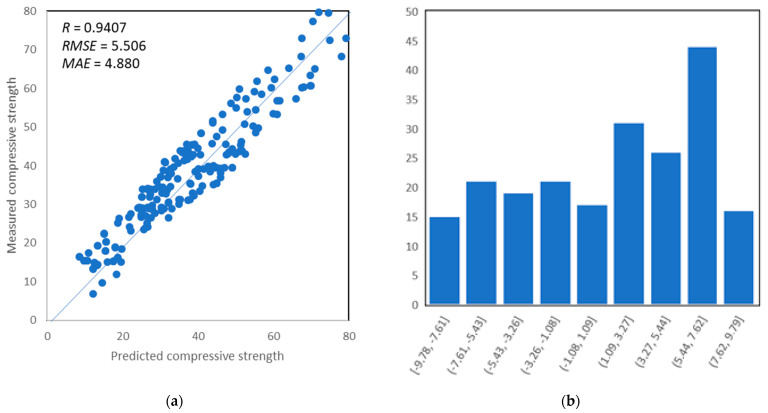

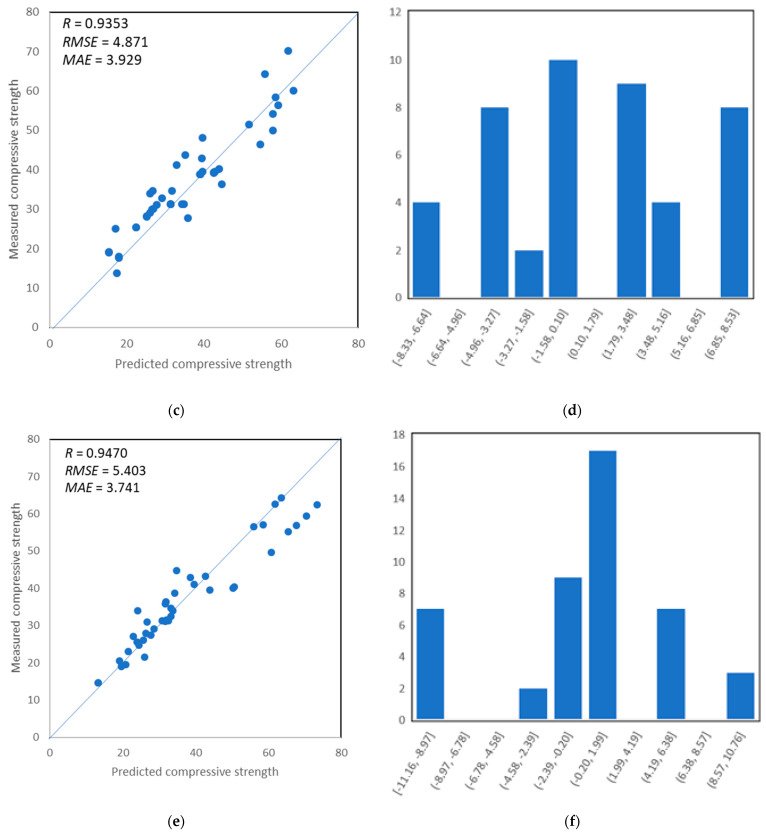
(**a**) Predictions vs. target data of training set; (**b**) error distribution of GEP model with prediction versus training set; (**c**) predictions vs. target data of validation set; (**d**) error distribution of GEP model with prediction versus validation set; (**e**) predictions vs. target data of testing set; (**f**) error distribution of GEP model with prediction versus testing set.

The evaluation of the model expectations against the actual results of SCC strength is graphically shown in Figure 10. It depicts that all input variables to predict *f*′*_c_* of SCC are accurately taken into account by the model. The presented results are highly correlated, as be seen in Figure 10a,c,e; it was also proved by the obtained values of linear correlation coefficient, equal to 0.941, 0.935, and 0.947 for training and validation. The proposed model’s efficiency is significantly affected by the number of datasets [63]. This research consists of 300 datasets in the prediction of SCC; hence, high accuracy of the model is expected. The response of predicted values with error distribution is presented in Figure 10b,d,f. It can be seen that all sets for the GEP model show a minimum error with the maximum range that lies below 10 MPa, as depicted in Figure 10b,d,f. It confirms the accuracy of the desired model with respect to regression models and it is on the same level of accuracy as for ANN.

### 5.4. Comparison between the Proposed Models

**Figure 11:**
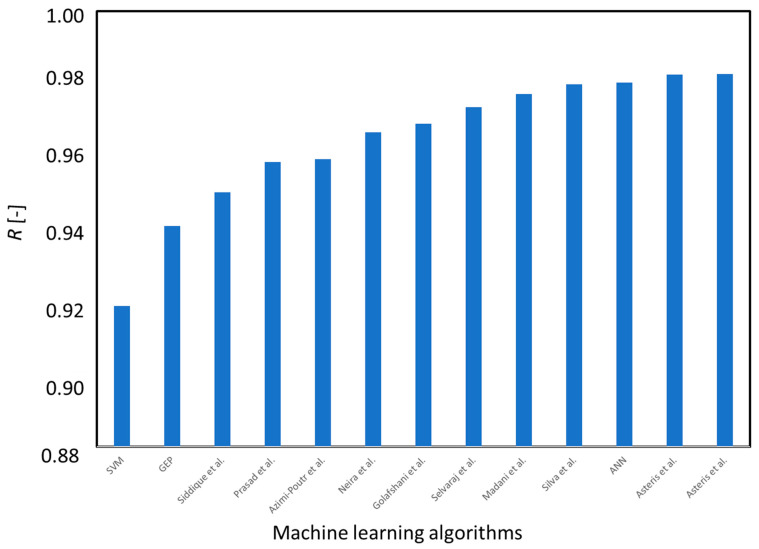
The comparison of models for SCC compressive strength prediction.

The machine learning algorithms used in the article are accurate in prediction of the compressive strength of self-compacting concrete modified by fly ash. It can be observed based on the values of the parameters describing their accuracy, which were linear coefficient of correlation *R*, root mean square error *RMSE*, and mean average error *MAE*. Among the artificial neural networks, the support vector machine and gene expression programming, there is difficult to point the most accurate algorithm. The least accurate was support vector machine due to the lowest values of the linear coefficient of correlation and the highest values of errors in all processes. However, even though the neural network was the most accurate during the training process, the gene expression programming algorithm was more accurate in the testing and validation processes. Thus, for construction practice, it might be beneficial to use this algorithm, which performs better in the testing and validation processes instead of training because of the threat of overfitting. In Figure 11, the aforementioned algorithms were compared with other models presented in the literature.

It can be seen that all of the investigated models are predict the SCC compressive strength well, according to the literature. However, due to the fact that none of the models were perfectly accurate (linear correlation coefficient was equal to 1.0), it is still possible to improve the algorithms by building other databases or using different algorithms.

## 6. Conclusions

This research discusses the machine learning application of artificial intelligence, in particular, artificial neural network, support vector machine, and gene expression programming for the prediction of self-compacting concrete compressive strength. By performing an extensive literature survey for obtaining the experimental results of the SCCs compressive strength values and also by performing numerical analysis using ANN, SVM, and GEP, the following conclusions can be drawn:

1. ANN-, SVM-, and GEP-based models predict the properties of SCC strength; however, ANN and GEP are the most accurate for this purpose;
2. ANN, SVM, and GEP models were characterized by the very high values of linear correlation coefficient equal to *R* = 0.9588, *R* = 0.9344 and *R* = 0.9353 for the testing set, respectively. The test set of the ANN, SVM, and GEP models show average error values of 5.428 MPa, 5.023 MPa, and 3.741 MPa, respectively. This indicates that the GEP model was able to be performed better in terms of accuracy during this process in comparison to the ANN and SVM models;
3. Permutation features show clear influential parameters for strength prediction. Variable such as the ratio of cement and fly ash added to the mixture have a major effect on strength with 53% out of total parameters. Thus, it is important to know their ratio in the mixture in order to evaluate the SCC compressive strength; without this variable, the modelling might be less accurate;
4. Statistical analysis and external checks give obstinate responses for all models.

These models were used for prediction rather than conducting experimental work; thus, their utilization in the civil engineering field will lower the carbon footprint. Below, a few recommendations for continuing similar research in the future are presented:

1. Hybrid models or advanced evolutionary algorithms can be developed, and the results can be compared to the present study.
2. The techniques used in this study can be used to model other engineering properties of concrete and structures.

As every study and technique has some limitations, some of the limitations of GEP are as follows:

1. Sometimes, the GEP is trapped in a local region that does not contain the global optimum. This phenomenon is called premature convergence and is one of the serious problems in genetic algorithms.
2. The “best” fitness is in comparison to other fitness; i.e., the stop criterion is not clear in every problem.
3. For specific optimization problems and problem instances, other optimization algorithms may be more efficient than genetic algorithms in terms of speed of convergence.

## Figures and Tables

**Table 1:** The latest works in the subject of concrete compressive strength prediction.

S. No	Algorithm Name	Notation	Dataset	Prediction Properties	Year	Waste Material Used	References
1	Artificial neural network	ANN	169	Compressive strength	2016	FA GGBFS SF RHA	[16]
2	Artificial neural network	ANN	205	Compressive strength	2019	FA GGBFS SF RHA	[17]
3	Artificial neural network	ANN	114	Compressive strength	2017	FA	[18]
4	Artificial neural network	ANN	80	Compressive strength	2011	FA	[19]
5	Artificial neural network	ANN	300	Compressive strength	2009	FA	[20]
6	Support vector machine	SVM	-	Compressive strength	2020	FA	[21]
7	Random forest	RF	131	Compressive strength	2019	FA GGBFS SF	[22]
8	Biogeographical-based programming	BBP	413	Elastic modulus	2016	SF FA SLAG	[23]
9	Intelligent rule-based enhanced multiclass support vector machine and fuzzy rules	IREMSVM-FR with RSM	114	Compressive strength	2019	FA	[24]
10	Support vector machine	SVM	115	Slump test L-box test V-funnel test Compressive strength	2020	FA	[25]
11	Multivariate adaptive regression spline	M5 MARS	114	Compressive strength Slump test L-box test V-funnel test	2018	FA	[26]

**Table 2:** Neural network properties.

Parameter	Neural Network Properties
Input parameters	Six (6)
Output parameters	One (1)
Percentage of training set/testing and validation set	70/30
Number of epochs	Hundred (100)
Performance limit	10^−6^
Training model	Supervised
Training process	Quasi-Newton
Activation function (HL)	Logistic (sigmoid)
Activation function (OL)	Logistic (linear)

**Table 3:** Range of input and output variables.

**Parameters**	**Minimum**	**Maximum**
**Input Variables**		
Cement (kg/m^3^)	83	540
Fly ash (kg/m^3^)	0	525
Coarse aggregate (kg/m^3^)	578	1125
Fine aggregate (kg/m^3^)	478	1180
Superplasticizer (%)	0	1.36
Water–binder ratio	0.22	0.9
**Output Variable**	**Minimum**	**Maximum**
Compressive strength (MPa)	8.54	78.4

**Table 4:** The dataset from the latest works in the subject of concrete compressive strength prediction.

Statistical Measures of Input Parameters and Output Strength in Modeling Prediction						
Dataset	Parameters					
**Training Set**	**Cement**	**Fly Ash**	**Water–Binder**	**Fine Aggregate**	**Coarse Aggregate**	**Superplasticizer**
Mean	292.48	118.08	0.48	802.90	912.45	0.18
Standard Error	6.69	6.13	0.01	6.76	7.82	0.02
Median	290.00	120.68	0.46	793.46	900.00	0.05
Mode	250.00	0.00	0.55	742.00	837.00	0.00
Standard Deviation	96.95	88.88	0.13	97.97	113.39	0.26
Sample Variance	9399.46	7899.70	0.02	9598.18	12,857.39	0.07
Kurtosis	−0.11	2.55	0.26	2.30	0.27	3.89
Skewness	0.51	0.87	0.76	0.57	−0.35	2.00
Range	457.00	525.00	0.67	693.00	547.00	1.36
Minimum	83.00	0.00	0.23	487.00	578.00	0.00
Maximum	540.00	525.00	0.90	1180.00	1125.00	1.36
Sum	61,421.24	24,796.10	100.81	168,608.70	191,614.79	37.16
Count	210.00	210.00	210.00	210.00	210.00	210.00
**Validation Set**	**Cement**	**Fly Ash**	**Water–Binder**	**Fine Aggregate**	**Coarse Aggregate**	**Superplasticizer**
Mean	292.65	117.73	0.49	789.71	912.83	0.18
Standard Error	13.13	13.29	0.02	15.47	18.82	0.04
Median	295.90	107.25	0.50	784.50	879.00	0.04
Mode	250.00	0.00	0.55	774.00	837.00	0.00
Standard Deviation	89.07	90.14	0.13	104.91	127.67	0.27
Sample Variance	7933.73	8125.93	0.02	11,005.42	16,300.13	0.07
Kurtosis	1.04	−1.12	−0.41	3.03	−0.04	4.61
Skewness	0.65	0.09	0.03	−0.08	−0.22	2.07
Range	396.40	275.00	0.58	657.00	535.00	1.25
Minimum	143.60	0.00	0.22	478.00	590.00	0.00
Maximum	540.00	275.00	0.80	1135.00	1125.00	1.25
Sum	13,461.90	5415.63	22.52	36,326.78	41,989.96	8.22
Count	46.00	46.00	46.00	46.00	46.00	46.00
**Test Set**	**Cement**	**Fly Ash**	**Water–Binder**	**Fine Aggregate**	**Coarse Aggregate**	**Superplasticizer**
Mean	292.76	101.57	0.49	837.83	912.01	0.15
Standard Error	12.53	11.32	0.02	13.30	20.65	0.03
Median	290.00	100.37	0.51	808.00	940.60	0.03
Mode	250.00	0.00	0.33	899.00	837.00	0.00
Standard Deviation	84.02	75.94	0.13	89.20	138.54	0.22
Sample Variance	7059.62	5767.29	0.02	7956.13	19,193.24	0.05
Kurtosis	−0.76	−0.93	−1.03	2.35	−0.75	1.99
Skewness	0.09	0.03	0.02	1.21	−0.45	1.66
Range	340.30	263.00	0.46	473.00	490.00	0.80
Minimum	134.70	0.00	0.27	662.00	621.00	0.00
Maximum	475.00	263.00	0.73	1135.00	1111.00	0.80
Sum	13,174.34	4570.57	22.21	37,702.21	41,040.48	6.82
Count	45.00	45.00	45.00	45.00	45.00	45.00

**Table 5:** Gene expression programming variables detail set.

**Settings**	
General property	${f^{\prime }}_{c}$
Chromosomes	30
Genes	3, 4, 5
Head size	8
Linking function	Multiplication
Function set	+, −, ×, ÷, exp
**Numerical Constants**	
Constant per gene	10
Data type	Floating number
Lower bound	−10
Upper bound	10
**Genetic Operators**	
Mutation rate	0.00138
Inversion rate	0.00546
Insertion Sequences transposition rate	0.00546
Root Insertion Sequence transposition rate	0.00546
One-point recombination rate	0.00277
Two-point recombination rate	0.00277
Gene recombination rate	0.00277
Gene transposition rate	0.00277

**Table 6:** Constant and notation used to prepare empirical equation.

Parameters Notation	Parameters	Constant Notations	Constant Values
d_0_	Cement	G1C5	−4.28835075
d_1_	Fly ash	G1C2	37.75001621
d_2_	Water–powder	G2C3	39.89209066
d_3_	Fine aggregate	G2C1	9.967413128
d_4_	Coarse aggregate	G2C5	26.22055325
d_5_	Superplasticizer	G3C9	−20.52776364
-	-	G3C7	145.5520044
-	-	G3C5	−10.5395382
-	-	G3C6	544.4511609

## Data Availability

All the data is available within the manuscript.

## Appendix A

**Table A1:** Self-compacting concrete mixture components and compressive strength.

No.	Cement	Fly Ash	Water–Powder Ratio	Sand	Coarse Aggregate	Superplasticizer	Strength
-	kg/m^3^	kg/m^3^	-	kg/m^3^	kg/m^3^	kg/m^3^	MPa
1	148	137	0.55	830	1002	0.11	17.95
2	393	0	0.49	758	940	0	39.58
3	325	60	0.65	900	850	0.12	31.4
4	374.3	0	0.51	730.4	1013.2	0.02	39.06
5	348	224	0.5	783	848	0.43	58.6
6	296	107	0.55	778	819	0.04	31.42
7	350	162	0.41	768	840	0.18	51.7
8	296	106.7	0.55	778.4	819.2	0.04	31.42
9	374	0	0.51	730	1013	0.02	39.05
10	148.1	136.6	0.56	830.1	1001.8	0.11	17.96
11	275	0	0.67	808	1088	0	24.5
12	231.75	121.62	0.49	778.45	1056.4	0.03	33.73
13	181.38	167.01	0.49	777.8	1055.6	0.04	27.77
14	194.68	100.52	0.56	905.9	1006.4	0.04	25.72
15	325	60	0.65	899	850	0.43	30.8
16	420	80	0.33	785	860	0.3	56
17	212.52	100.37	0.51	903.59	1007.8	0.04	31.64
18	290	100	0.33	913	837	0.01	42.7
19	333	0	0.58	842.6	931.2	0	31.97
20	250	160	0.55	742	837	0.5	28.5
21	339	0	0.58	781	968	0	32.04
22	207	207	0.45	845	843	0.4	33.2
23	252	0	0.73	784	1111	0	19.69
24	360	240	0.28	853	698	0.3	63.5
25	417	153	0.32	828	759	0.31	61.82
26	163	245	0.4	851	851	0.2	26.2
27	310	0	0.62	850	970	0	27.92
28	350	133	0.38	815	883	0.34	55.3
29	190.34	125.18	0.51	802.59	1088.1	0.05	28.47
30	427	115	0.36	779	844	0.26	59.4
31	475	0	0.48	594	932	0	39.29
32	164.6	150.4	0.58	728.9	1023.3	0.07	18.03
33	165	150	0.58	729	1023	0.07	18.03
34	318	126	0.47	737	861	0.02	40.06
35	317.9	126.5	0.47	736.6	860.5	0.02	40.06
36	280	96	0.87	817	850	0.62	15.9
37	540	0	0.32	613	1125	0	67.31
38	183	160	0.55	891	837	0.5	22.1
39	238.05	94.11	0.56	847.01	949.91	0.03	30.23
40	307	0	0.63	812	968	0	27.53
41	480	96	0.38	819	699	0.94	53
42	144.8	133.6	0.65	811.5	979.5	0.08	13.2
43	145	134	0.65	812	979	0.08	13.2
44	151.6	111.9	0.7	815.9	992	0.05	12.18
45	325	60	0.85	722	850	0.43	13.3
46	370	24	0.69	772	850	0.25	26.4
47	152	112	0.7	816	992	0.05	12.18
48	331	0	0.58	825	978	0	31.45
49	252.5	0	0.74	784.3	1111.6	0	19.77
50	505	60	0.35	630	1030	0	64.02
51	134.7	165.7	0.6	804.9	961	0.07	13.29
52	325	60	0.65	899	850	0.43	32.6
53	135	166	0.6	805	961	0.07	13.29
54	475	0	0.34	662	1044	0.02	58.52
55	251.37	118.27	0.52	754.3	1043.6	0.02	33.27
56	166.09	163.27	0.54	780.09	1058.6	0.03	21.54
57	393	0	0.49	785.6	940.6	0	39.6
58	250	0	0.73	820	1100	0	20.87
59	210	100	0.65	910	837	0.8	19.1
60	190.68	125.4	0.51	804.01	1090	0.04	26.4
61	249	60	0.68	1079	850	0.43	24
62	405	0	0.43	695	1120	0	52.3
63	528	0	0.35	720	920	0.01	56.83
64	250	160	0.55	739	837	0	27.3
65	273	90	0.55	762	931	0.04	32.24
66	297.16	117.54	0.42	753.45	1022.8	0.03	47.4
67	169	254	0.45	853	853	0	30.2
68	272.6	89.6	0.55	762.2	931.3	0.04	32.25
69	190.34	125.18	0.53	798.9	1079	0.05	24.85
70	310	0	0.62	830	1012	0	27.83
71	298	107	0.52	744	880	0.04	31.87
72	298.2	107	0.52	744.2	879.6	0.04	31.88
73	251.37	118.27	0.51	757.73	1028.4	0.03	32.66
74	247	165	0.45	845	846	0.12	34.6
75	400	0	0.47	745	1025	0	43.7
76	170	200	0.43	930	900	0.2	31
77	289	0	0.66	895.3	913.2	0	25.57
78	317	160	0.55	594	837	0.5	29.1
79	326	138	0.43	792	801	0.03	40.68
80	295	0	0.63	769	1069	0	25.18
81	520	0	0.33	855	855	0.01	60.28
82	225	275	0.35	908	652	0.7	41.42
83	222.36	96.67	0.59	870.32	967.08	0.02	24.89
84	165	143.57	0.53	900.9	1005.6	0	26.2
85	238	0	0.78	789	1119	0	17.54
86	238	0	0.78	789	1118	0	17.54
87	238.1	0	0.78	789.3	1118.8	0	17.58
88	238	159	0.4	844	844	0.29	37.8
89	522	0	0.28	896	896	0	74.99
90	148	182	0.55	884	839	0.1	15.52
91	290	100	0.65	709	837	0.2	26.6
92	148.1	182.1	0.55	884.3	838.9	0.1	15.53
93	154.8	142.8	0.65	696.7	1047.4	0.06	12.46
94	302	0	0.67	817	974	0	21.75
95	155	143	0.65	697	1047	0.06	12.46
96	280	120	0.39	946	900	0.35	45
97	255	0	0.75	945	889.8	0	18.75
98	250	160	0.55	746	837	1	26.7
99	400	60	0.63	718	850	0.43	30.4
100	322	0	0.63	800	974	0	25.18
101	250	160	0.55	742	837	0.5	26.4
102	220	180	0.45	850	900	0.35	38
103	350	90	0.48	852	923	0.14	46.5
104	290	100	0.45	913	837	0.8	42.7
105	213.74	174.74	0.4	776.35	1053.5	0.05	40.15
106	331	0	0.58	821	1025	0	31.74
107	427	115	0.45	779	844	0.12	59.4
108	295.8	0	0.63	769.3	1091.4	0	25.22
109	281	0	0.66	774	1104	0	22.44
110	281	0	0.66	774	1104	0	22.44
111	296	0	0.63	769	1090	0	25.18
112	325	60	0.65	898	850	0.43	34.3
113	250	160	0.55	742	837	0.5	26
114	275	275	0.37	796	937	0.74	63.32
115	300	0	0.61	795	1075	0	26.85
116	298.1	107	0.46	815.2	879	0.02	42.64
117	298	107	0.46	815	879	0.02	42.64
118	290	100	0.48	709	837	0	26.6
119	325	60	0.65	896	850	0.75	27.7
120	220	180	0.39	916	900	0.6	43
121	370	96	0.57	833	850	0.25	39.5
122	225	0	0.8	833	1113	0	17.34
123	200	200	0.4	842	843	0.17	34.9
124	143.6	174.9	0.5	844.5	942.7	0.12	15.42
125	250	95.69	0.54	861.17	956.86	0.02	29.22
126	144	175	0.5	844	943	0.13	15.42
127	322	138	0.35	693.81	1085.2	0	58
128	322.5	107.5	0.47	1135	630	0.01	43.98
129	325	60	0.65	898	850	0.43	35
130	250	160	0.55	742	837	0.5	25.3
131	212.07	121.62	0.54	779.32	1057.6	0.03	24.9
132	301	129	0.47	1135	630	0.01	44
133	325	0	0.57	783	1063	0	30.57
134	218.85	124.13	0.46	794.91	1078.7	0.05	30.22
135	325	60	0.65	899	850	0.43	35.3
136	370	96	0.57	830	850	0.62	38.8
137	170	200	0.43	928	900	0.5	33
138	330	220	0.32	700	899	0.69	60.9
139	375	0	0.5	758	1038	0	38.21
140	275	250	0.35	775	840	0.2	54.5
141	399	100	0.35	814	882	0.15	55
142	339	0	0.55	754	1060	0	31.65
143	233.81	94.58	0.6	852.16	947.04	0.02	22.84
144	326.5	137.9	0.43	792.5	801.1	0.03	38.63
145	210	220	0.45	768	837	0.8	26.7
146	277	0	0.69	856	968	0	25.97
147	350	0	0.53	770	1050	0	34.29
148	339.2	0	0.55	754.3	1069.2	0	31.9
149	339	0	0.55	754	1069	0	31.84
150	220	180	0.39	916	900	0.1	44
151	280	96	0.87	820	850	0.25	19.6
152	350	150	0.35	900	600	1	37.18
153	229.68	118.16	0.56	757.63	1028.1	0.03	24.54
154	237	133	0.36	1034	900	0.2	49
155	258	172	0.47	1135	630	0.01	43.18
156	150.9	183.9	0.5	772.2	991.2	0.08	15.57
157	295.71	95.64	0.44	859.2	955.14	0.03	39.94
158	151	184	0.5	772	991	0.08	15.57
159	300	300	0.28	787	720	0.33	52.7
160	220	330	0.32	686	881	0.62	47.5
161	250	95.69	0.55	857.2	948.9	0.02	27.22
162	275	155	0.43	827	900	0.5	48
163	277.05	97.39	0.43	875.61	973.9	0.04	48.28
164	229.97	118.31	0.56	758.59	1029.4	0.02	24.48
165	165	385	0.34	656	834	1	34.9
166	145	179	0.62	869	824	0.06	10.54
167	327	173	0.35	902	803	0.41	61.6
168	279.5	150.5	0.47	1135	630	0.01	44.34
169	145.4	178.9	0.62	868.7	824	0.05	10.54
170	83	468	0.41	624	794	1	14.64
171	325	325	0.34	611	777	1.18	50.07
172	376	0	0.57	762.36	1003.5	0	31.97
173	251.37	118.27	0.51	757.73	1028.4	0.02	29.65
174	250	160	0.55	742	837	0.5	24.1
175	250	160	0.38	919	837	0.5	36.3
176	290	220	0.45	625	837	0.2	32.9
177	296	0	0.65	765	1085	0	21.65
178	428	257	0.27	788	736	0.02	74.5
179	250	257	0.38	787	853	0.23	51.5
180	350	0	0.58	775	974	0	27.34
181	200	0	0.9	845	1125	0	12.25
182	183	160	0.29	891	837	0.01	22.1
183	220	180	0.39	916	900	0.35	45
184	212	124.78	0.47	799.54	1085.4	0.04	38.5
185	250	160	0.34	742	837	0.01	28.5
186	500	0	0.28	853	966	0.01	67.57
187	325	120	0.75	755	850	0.43	32.2
188	154.8	142.8	0.65	867.7	877.2	0.06	9.74
189	155	143	0.65	868	877	0.06	9.74
190	500	101	0.32	820	753	0.38	70.93
191	382	0	0.49	739	1047	0	37.42
192	150.7	185.3	0.5	678	1074.5	0.1	13.46
193	382.5	0	0.49	739.3	1047.8	0	37.44
194	151	185	0.5	678	1074	0.11	13.46
195	225	525	0.33	487	620	1.36	34.83
196	275.07	121.35	0.4	777.5	1053.6	0.04	51.33
197	349	0	0.55	809	1056	0	33.61
198	313	113	0.42	689	1002	0.03	36.8
199	313.3	113	0.42	688.7	1001.9	0.03	36.8
200	348	224	0.31	783	848	0.9	58.6
201	420	180	0.32	900	750	0.03	79.19
202	276	184	0.35	693.81	1085.2	0	56
203	385	136	0.3	768	903	0.05	55.55
204	382	0	0.48	739	1047	0	37.42
205	440	110	0.32	714	917	0.69	69.8
206	349	162	0.39	779	852	0.29	59.9
207	349	0	0.55	806	1047	0	32.72
208	477	53	0.45	768	668	0.09	32.19
209	212.57	100.39	0.51	903.79	1003.8	0.05	37.4
210	325	0	0.55	1042	850	0.43	41.2
211	397	0	0.47	734	1040	0	39.09
212	250	160	0.72	566	837	0.5	11
213	370	24	0.69	770	850	0.62	18.7
214	236	0	0.82	885	968	0	18.42
215	250	160	0.34	739	837	0	27.3
216	197	197	0.35	856	856	0.28	38.9
217	237	133	0.43	960	900	0.5	46
218	350	133	0.52	815	883	0.16	55.3
219	165	385	0.58	735	865	0.84	37.92
220	312.7	0	0.57	822.2	999.7	0.03	25.1
221	317	160	0.37	594	837	0.01	29.1
222	313	0	0.57	822	1000	0.03	25.1
223	350	90	0.39	852	923	0.3	46.5
224	313.8	112.6	0.4	782.9	925.3	0.03	38.46
225	380	20	0.38	1180	578	0.4	40.4
226	407	244	0.28	815	761	0.02	70.4
227	314	113	0.4	783	925	0.03	38.46
228	248	203	0.39	808	900	0.35	50
229	304.8	99.6	0.48	705.2	959.4	0.03	30.12
230	305	100	0.48	705	959	0.03	30.12
231	480	0	0.4	712.2	936.2	0	43.94
232	220	180	0.33	982	900	0.35	51
233	164	200	0.5	846	849	0.08	15.09
234	210	100	0.44	910	837	0.01	19.1
235	344	147	0.35	814	881	0.12	48.75
236	164.2	200.1	0.5	846	849.3	0.08	15.09
237	357	193	0.33	878	742	0.02	67.5
238	275	155	0.43	830	900	0.2	36
239	333	215	0.33	835	766	0.24	50.24
240	220	180	0.39	916	900	0.35	47
241	250	160	0.34	746	837	0.01	26.7
242	321	128	0.41	780	870	0.03	37.26
243	321.4	127.9	0.41	779.7	870.1	0.04	37.27
244	250	160	0.23	919	837	0.01	36.3
245	250	160	0.34	742	837	0.01	26.4
246	355.9	141.6	0.39	778.4	801.4	0.03	40.87
247	356	142	0.39	778	801	0.03	40.87
248	460	0	0.35	693.81	1085.2	0	68
249	298	107	0.4	784	953	0.04	35.86
250	350	111	0.39	831	900	0.32	61
251	485	0	0.3	800	1120	0	71.99
252	298.1	107.5	0.4	784	953.2	0.04	35.87
253	480	0	0.4	721	936	0	43.89
254	198	232	0.34	874	900	0.2	46
255	158	195	0.62	713	898	0.07	8.54
256	350	162	0.59	768	840	0.09	51.7
257	158.4	194.9	0.62	712.9	897.7	0.07	8.54
258	251.81	99.94	0.42	899.76	1006	0.05	33.94
259	249.1	98.75	0.45	889.01	987.76	0.05	30.85
260	210	220	0.22	786	837	0.01	26.7
261	275	275	0.34	691	880	1.25	57.9
262	250	160	0.34	742	837	0.01	26
263	250	261	0.55	478	837	0.5	17
264	161	241	0.35	866	864	0.3	35.8
265	300	200	0.35	923	663	0.7	54.69
266	540	0	0.3	676	1055	0	61.89
267	290	220	0.26	625	837	0	32.9
268	525	0	0.36	613	1125	0	55.94
269	213.5	174.24	0.41	771.9	1043.6	0.05	44.64
270	465	85	0.41	910	590	0.02	35.19
271	250	275	0.34	842	772	0.23	39.62
272	210	220	0.65	562	837	0.2	10.2
273	397	0	0.47	734	1040	0	36.94
274	368	92	0.35	693.81	1085.2	0	66
275	465	85	0.41	910	590	0.97	35.19
276	250	160	0.34	742	837	0.01	25.3
277	520	0	0.34	805	870	0.01	51.02
278	213.5	174.24	0.4	775.48	1052.3	0.05	45.94
279	193	158	0.39	1024	900	0.35	44
280	437	80	0.34	743	924	0.43	69.7
281	500	0	0.3	655	1033	0.02	69.84
282	336.5	0	0.54	816.8	985.8	0.01	44.87
283	336	0	0.54	817	986	0.01	44.86
284	220	180	0.39	916	900	0.35	49
285	246.83	125.08	0.39	800.89	1086.8	0.05	52.5
286	220	180	0.39	916	900	0.12	49
287	385	0	0.48	763	966	0	31.35
288	540	60	0.33	900	750	0.02	78.05
289	322.2	115.6	0.45	813.4	817.9	0.03	31.18
290	322	116	0.45	813	818	0.03	31.18
291	250	160	0.34	742	837	0.01	24.1
292	290.35	96.18	0.43	865	961.18	0.03	34.74
293	252.31	98.75	0.42	889.01	987.76	0.06	50.6
294	380	145	0.35	988	659	0.28	65.5
295	344	86	0.47	1135	630	0.01	50.37
296	438	263	0.27	774	723	0.02	69.5
297	380	192	0.35	931	621	0.21	67.8
298	412	138	0.33	887	752	0.02	73.4
299	350	186	0.33	786	851	0.22	70.4
300	375	125	0.35	938	673	0.7	60.8
